# 2.4 GHz GaN HEMT Class-F Synchronous Rectifier Using an Independent Second Harmonic Tuning Circuit

**DOI:** 10.3390/s21051608

**Published:** 2021-02-25

**Authors:** Jongyun Na, Sang-Hwa Yi, Jaekyung Shin, Hyungmo Koo, Jongseok Bae, Keum-Cheol Hwang, Kang-Yoon Lee, Youngoo Yang

**Affiliations:** 1 College of Information and Communication Engineering, Sungkyunkwan University, 2066 Seobu-ro, Jangan-gu, Suwon 16419, Korea; realiuz@naver.com (J.N.); shaq2442@gmail.com (J.S.); 99hyungmo@gmail.com (H.K.); baeyas0@gmail.com (J.B.); khwang@skku.edu (K.-C.H.); klee@skku.edu (K.-Y.L.); 2 Electrical Environment Research Center, Korea Electrotechnology Research Institute, Changwon-si 51543, Korea; shyi@keri.re.kr

**Keywords:** wireless power transmission, RF synchronous rectifier, class-F power amplifier, GaN HEMT device, time reversal duality, independent harmonic tuning circuit

## Abstract

This paper proposes a class-F synchronous rectifier using an independent second harmonic tuning circuit for the power receiver of 2.4 GHz wireless power transmission systems. The synchronous rectifier can be designed by inverting the RF output port to the RF input port of the pre-designed class-F power amplifier based on time reversal duality. The design of the class-F power amplifier deploys an independent second harmonic tuning circuit in the matching networks to individually optimize the impedances of the fundamental and the second harmonic. The synchronous rectifier at the 2.4 GHz frequency is designed and implemented using a 6 W gallium nitride high electron mobility transistor (GaN HEMT). Peak RF-dc conversion efficiency of the rectifier of 69.6% is achieved with a dc output power of about 7.8 W, while the peak drain efficiency of the class-F power amplifier is 72.8%.

## 1. Introduction

As interest increases in wireless power transfer techniques and RF energy harvesting systems for various mobile/wearable or wireless sensor applications, highly efficient RF-dc converters or rectifiers are rapidly growing in importance [1,2,3,4]. Rectifiers can be classified into two types: (1) rectifiers based on a Schottky diode [5,6]; and (2) synchronous rectifiers based on a switched transistor [7,8]. While diode rectifiers have simpler structure than the synchronous rectifiers, synchronous rectifiers have higher power handling capability, especially for RF applications [9]. The high-power GaN HEMT [10] can be an effective choice for the synchronous rectifier design, due to its high-power and high-frequency switching capabilities (from a few Watts to a few hundred Watts and from a few MHz ranges to several GHz ranges, respectively) [11].

A synchronous rectifier can be designed by inverting the RF output port to the RF input port of the RF power amplifier circuit based on the time reversal duality [12]. The dc output of the rectifier can then be obtained from the load in the dc supply network to the drain of the power amplifier. To convert the power amplifier to the rectifier, the RF signal should be applied to the gate of the transistor for synchronous switching. To provide the gate with an RF signal, the feedback capacitance, $C_{gd}$, of the transistor can be used to couple the signal from the drain to the gate [13]. The RF signal can be tapped by a coupler, and, after controlling its phase using a phase shifter, it can be supplied to the gate [14].

For power amplifiers, harmonic control is important to achieve high efficiency with high RF output power [15]. Class-F power amplifiers, which have short circuits for the even-order harmonics and open circuits for the odd-order harmonics, have been a good option to obtain high efficiency [16]. An independent second-harmonic control circuit using quarter-wave transmission lines was proposed for a harmonic tuned power amplifier [17]. Since the independent second-harmonic tuning circuit does not affect the impedance matching circuit for the fundamental while tuning the second harmonic, it makes it much easier to tune the overall circuits for the second harmonic and the fundamental, and to produce optimal performances.

Several high-efficiency synchronous rectifiers have been reported using GaN HEMTs [18,19,20]. Ref. [18] reported a harmonic controlled class-F rectifier at the 0.985 GHz frequency with an RF-dc conversion efficiency of 81.3%. Ref. [19] reported a class-$F^{-1}$ wideband rectifier for the 0.6–1.15 GHz frequency range with an RF-dc conversion efficiency of 80.1%. Ref. [20] reported a class-$F^{-1}$ rectifier using a dc polarity control at the 1.8 GHz frequency with an RF-dc conversion efficiency of 77%. These previous works employed an external coupler and phase shifter to supply the RF signal to the gate. They also employed harmonic control circuits that could not be tuned without disturbing the fundamental impedance of the matching network.

In this work, we propose a class-F synchronous rectifier employing an independent second harmonic tuning circuit that uses a GaN HEMT with integrated coupler and phase shifter on a module. The independent second harmonic tuning circuit was designed for class-F operation in the 2.4 GHz band. With the ability to independently tune the fundamental and second harmonic impedances of the proposed circuits, it becomes easier to experimentally achieve high efficiency and high output power of the power amplifier and the rectifier, where fine tuning after implementation is inevitable, due to the error in the large-signal model of the transistor, and inaccuracy in the RF simulation of the passive circuits. Experimental results are presented and compared to the results of previous work.

## 2. The Independent Second Harmonic Tuning Circuit

**Figure 1:**
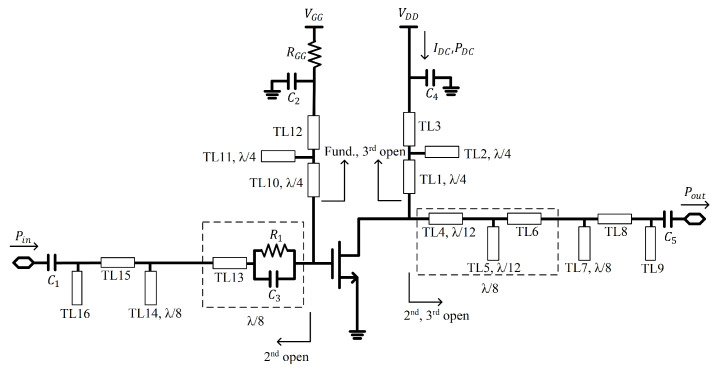
Schematic of the proposed class-F power amplifier using an independent second harmonic tuning circuit.

Figure 1 shows a schematic of the proposed class-F power amplifier using the independent harmonic tuning circuits at both the input and output networks. In the output matching network of the power amplifier, to separate the third harmonic tuning circuit from the fundamental and second harmonic matching circuits and to realize the optimized third harmonic load impedance for class-F operation, an optimized length of the series transmission line (TL4) and a quarter-wave open stub (TL5) at the third harmonic frequency are deployed before the fundamental matching network. To independently tune the second harmonic impedance, a transmission line (TL6) is added after TL4 and TL5 to make the overall length of the lines, TL4, TL5, and TL6, a quarter-wavelength at the second harmonic frequency. After TL6, a quarter-wave open stub (TL7) at the second harmonic frequency is added for the second harmonic load impedance to be open-circuited. Similarly, at the input network, a series line of TL13 and a parallel RC network using $R_{1}$ and $C_{3}$ should be designed to have a quarter-wavelength at the second harmonic frequency. A quarter-wave open stub (TL14) at the second harmonic frequency should come for the second harmonic source impedance to be open-circuited. A resistor $R_{GG}$ on the bias line and a parallel network with a resistor $R_{1}$ and a capacitor $C_{3}$ on the input matching network are used to improve the stability of the power amplifier.

For independent tuning of the second harmonic impedance and bias feeding, a series quarter-wave line (TL1 at the output and TL10 at the input), a quarter-wave open stub (TL2 at the output and TL11 at the input), and a series tuning line (TL3 at the output and TL12 at the input) are used at both the input and output matching networks. Using a series quarter-wave line and a quarter-wave open stub for the fundamental frequency, the fundamental impedance can be allowed to be open-circuited. Since quarter-wave lines at the fundamental frequency become half-wave lines at the second harmonic frequency, they do not affect the second harmonic impedances. Then, the second harmonic impedances at the input and output can be independently tuned using TL3 and TL12, by tuning the positions of the RF bypass capacitors of $C_{2}$ and $C_{4}$, respectively. The fundamental impedances at the input and output can be matched using matching networks based on a series transmission line (TL15 at the input and TL8 at the output) and an open stub (TL16 at the input and TL9 at the output), respectively.

**Figure 2:**
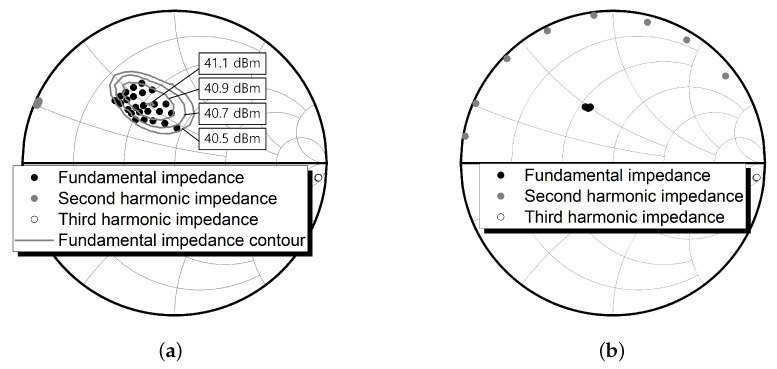
Load impedance tuning: (**a**) fundamental impedance tuning using TL8 and TL9; and (**b**) second harmonic impedance tuning using TL3.

Figure 2 shows the simulated load impedances for the fundamental impedance tuning (in Figure 2a) and for the second harmonic tuning (in Figure 2b). For the fundamental impedance tuning, the lengths of TL8 and TL9 were tuned from 0.08 to 0.12 λ and from 0.12 to 0.16 λ, respectively. Figure 2a shows that the second and third harmonic impedances are in almost fixed positions, while the fundamental impedances have wide variation. Otherwise, for the second harmonic impedance tuning using the length of TL3 from 0.03 to 0.1 λ for the second harmonic frequency, a wide variation of the second harmonic impedances with almost fixed positions for the fundamental and third harmonic impedances can be found (see Figure 2b).

**Figure 3:**
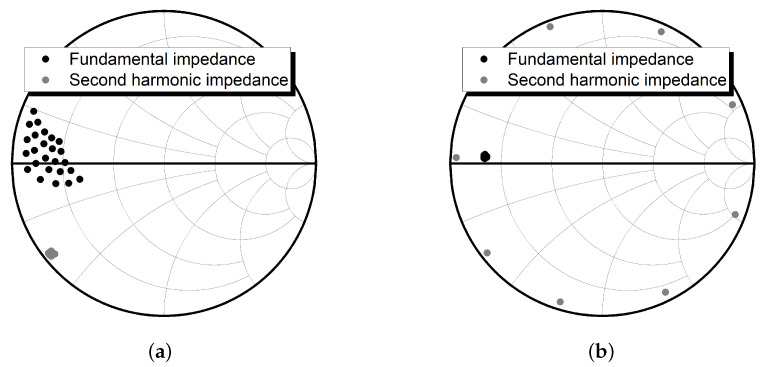
Source impedance tuning: (**a**) fundamental impedance tuning using TL15 and TL16; and (**b**) second harmonic impedance tuning using TL12.

Figure 3 shows the simulated source impedances for the fundamental impedance tuning (Figure 3a) and for the second harmonic tuning (Figure 3b). For the fundamental impedance tuning, the lengths of TL15 and TL16 were tuned from 0.29 to 0.37 λ and from 0.12 to 0.2 λ, respectively. Figure 3a shows that the second harmonic impedances are in almost fixed position, while the fundamental impedances have wide variation. Otherwise, for the second harmonic impedance tuning using the length of TL12 from 0.3 to 0.5 λ for the second harmonic frequency, a wide variation of the second harmonic impedances with an almost fixed position for the fundamental impedances can be found (see Figure 3b).

**Figure 4:**
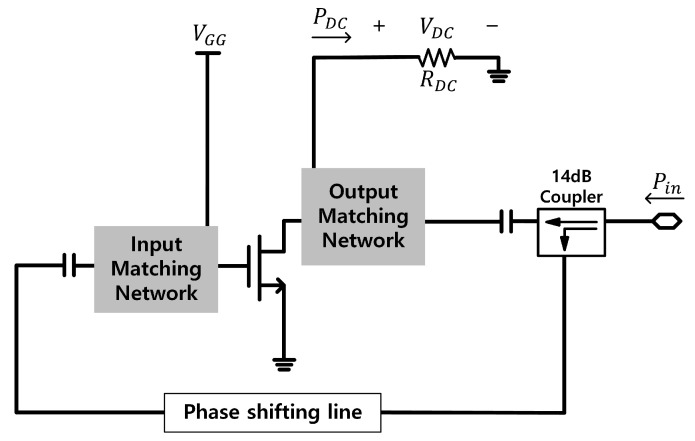
Schematic of the proposed class-F rectifier using the independent second harmonic tuning circuit based on time reversal duality.

Based on the time reversal duality, the rectifier circuit can be obtained from the power amplifier by inverting the RF output port of the amplifier to the RF input port of the rectifier. Figure 4 shows a schematic of the synchronous rectifier. The drain dc supply for the power amplifier becomes a dc load ($R_{DC}$) of the rectifier. Theoretically, $R_{DC}$ should have the value $V_{DD}$/$I_{DC}$ of the power amplifier in large signal condition. However, due to the loss of circuits and the error in the device model, the value of $R_{DC}$ needs to be refined to achieve high performances. To provide the RF signal to the gate, a coupler and a phase shifter are placed on the module. The coupling factor of the coupler is 14 dB, which should be almost equal to the gain of the power amplifier. The phase of the RF signal in the gate needs to be adjusted for high RF-dc conversion efficiency using the phase shifter, which is a phase shifting transmission line on the module.

## 3. Implementation and Experimental Results

**Figure 5:**
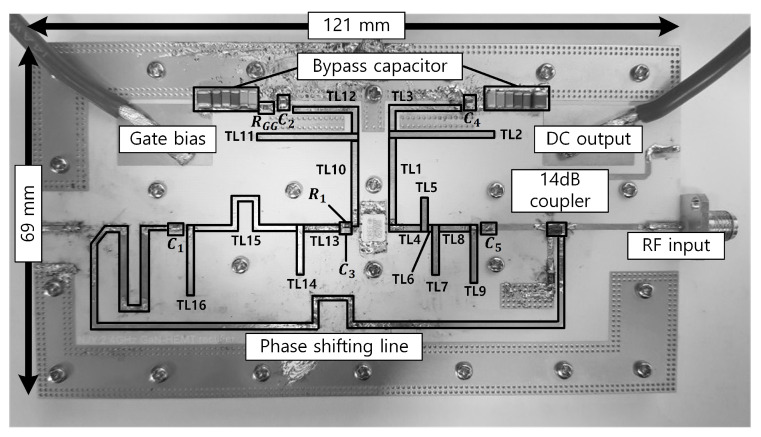
Photograph of the fabricated synchronous rectifier module.

Figure 5 shows a photograph of the fabricated synchronous rectifier module. The module was implemented using a Cree GaN HEMT (CGH40006P) and a printed circuit board (PCB) based on a Rogers 4350B substrate with a thickness of 20 mil and a dielectric constant of 3.66. The dimensions of the overall module are 121 mm × 69 mm. An AVX CP0805B2442BW directional coupler is used to couple the signal. The drain and gate supply voltages are set to 28 and –2.6 V, respectively.

**Figure 6:**
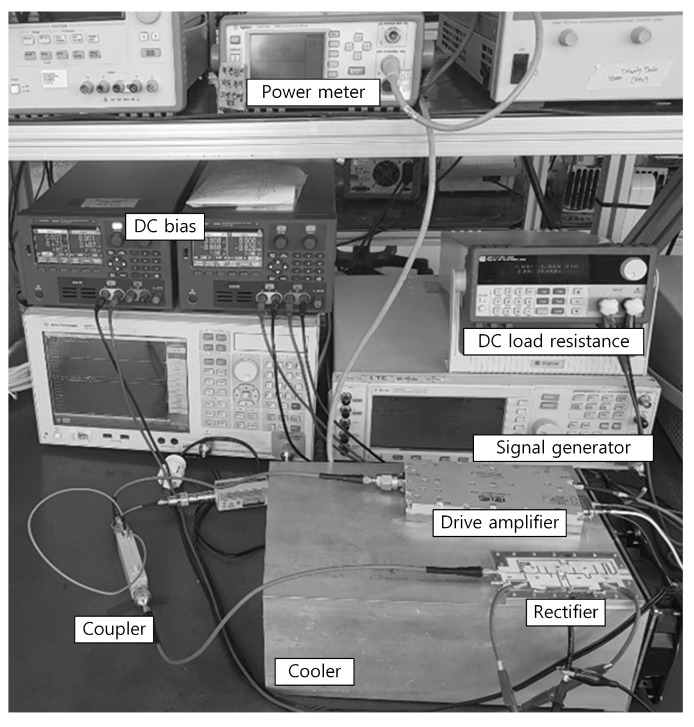
Photograph of the test bench for the synchronous rectifier.

Figure 6 shows a photograph of the test bench. An RFHIC RWP2060050-48 external drive amplifier is used to drive the input RF signal for the test. An RF-Lambda RFCD9M9G35 external directional coupler with a power meter is used to observe and to adjust the input RF power. The dc output port of the rectifier is connected to an electrical load to measure the delivered dc power to the load.

**Figure 7:**
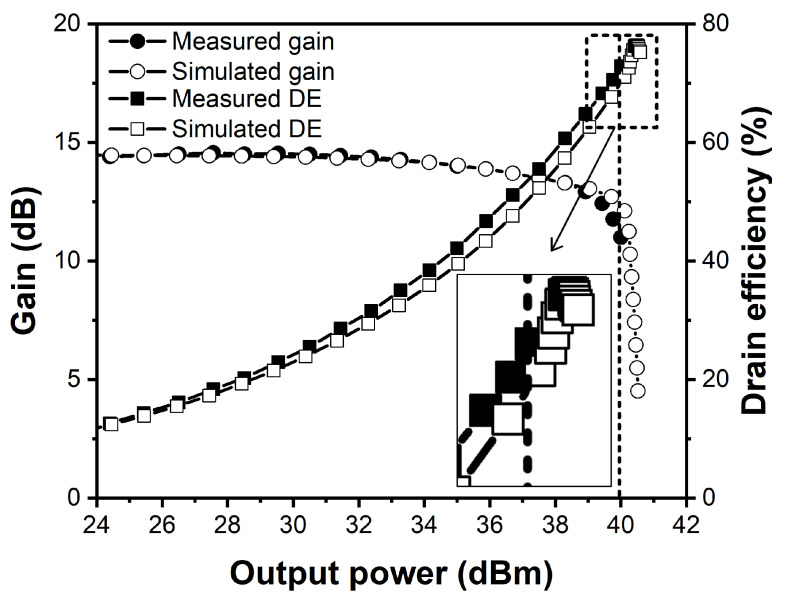
Simulated and measured gain and drain efficiency of the class-F power amplifier as a function of the output power at 2.4 GHz.

Figure 7 shows simulated and measured results of the class-F power amplifier. After implementation, the power amplifier was additionally optimized by independently tuning the fundamental and second harmonic impedances. The measured drain efficiency and gain of the fabricated power amplifier are 72.8% and 11 dB at the peak output power of 40 dBm, respectively. The simulated and measured performances show good agreement.

**Figure 8:**
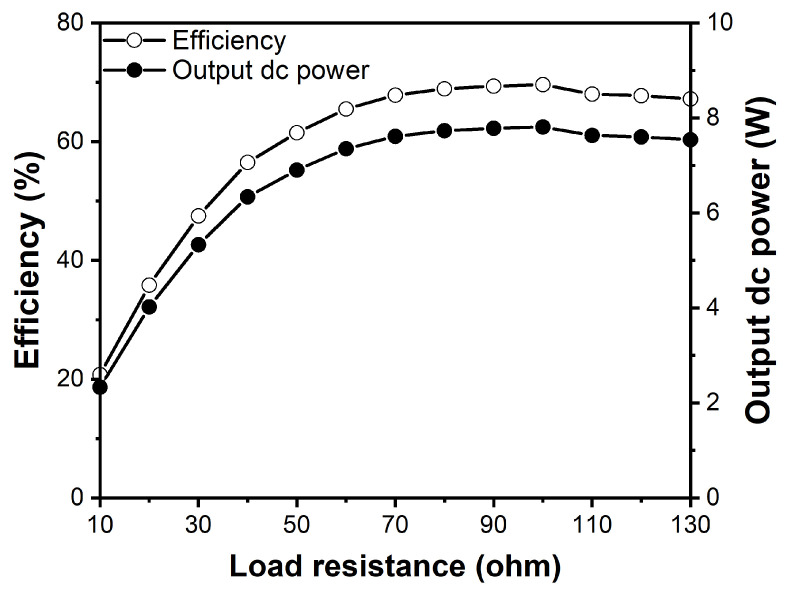
Measured RF-dc conversion efficiency and output dc power of the synchronous rectifier as a function of load resistance.

Figure 8 presents the measured RF-dc conversion efficiency and the dc power according to the load resistance values. The RF-dc conversion efficiency is obtained as follows.

(1) $$\mathrm{RF}-\mathrm{dc}\;\mathrm{conversion}\;\mathrm{efficiency}=\frac{P_{DC}}{P_{in}},$$

where $P_{in}$ is an input RF power and $P_{DC}$ is an output dc power delivered to the load resistance of $R_{DC}$. $P_{DC}$ is obtained using the dc voltage at the load resistance as follows.

(2) $$P_{DC}=\frac{{\left(V_{DC}\right)}^{2}}{R_{DC}}.$$

The measurements were done at the 2.4 GHz frequency and 11.22 W RF input power. Figure 8 shows that the optimum load resistance is about 100 Ω, where an RF-dc conversion efficiency of 69.6% and an output dc power of 7.81 W were obtained. From Equation (1), it can be found that the RF-dc conversion efficiency and the output dc power should be directly proportional when the RF input power is fixed.

**Figure 9:**
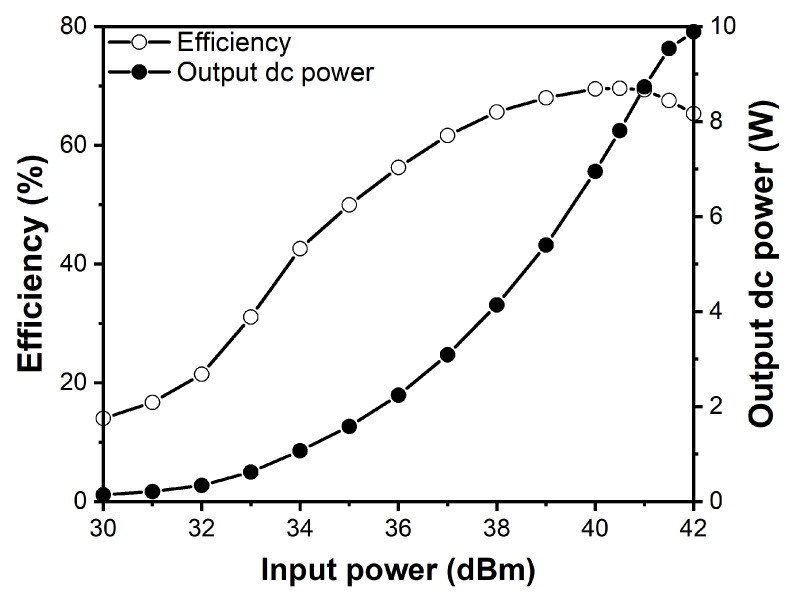
Measured power efficiency and output dc power according to the input power.

Figure 9 shows the RF-dc conversion efficiency and output dc power of the synchronous rectifier according to the input power at the 2.4 GHz frequency with the 100 Ω load resistance. Considering the loss of the coupler and the phase shifting lines that are added to the rectifier, more RF input power of the rectifier should be supplied than the RF output power of the class-F power amplifier. An RF-dc conversion efficiency of 69.6% for an 11.22 W (40.5 dBm) input power was achieved. The RF-dc conversion efficiency was sustained above 50% for input power level of no less than 35 dBm, and the maximum dc power obtained was 9.89 W with an efficiency of 65.3%.

Table 1 summarizes the performances of the implemented synchronous rectifier and compares them to the previous works. This work achieved a relatively high output dc power (7.81 W) with very high RF-dc conversion efficiency in the relatively higher 2.4 GHz frequency, using a transistor with a relatively low power capacity (6 W). In contrast, the authors of [18,19,20,21] used a transistor with a relatively higher output power capacity (10 W).

## 4. Conclusions

A class-F synchronous rectifier with an independent second harmonic tuning circuit was designed and implemented at the 2.4 GHz frequency. The synchronous rectifier was implemented by reconfiguring the class-F power amplifier based on the time reversal duality. It also includes a coupler and a phase shifting line that are integrated on the module. The peak drain efficiency of the fabricated class-F power amplifier was 72.8%, while the peak RF-dc conversion efficiency of the synchronous rectifier was 69.6% with a high dc output power of about 7.8 W using a transistor with a relatively low power capacity, compared to the previous works. The maximum dc output power of the rectifier was 9.89 W with an RF-dc conversion efficiency of about 65.3%. Since the size of the synchronous rectifiers is generally larger than that of the diode rectifiers, one of the future research topics could be size reduction of the synchronous rectifier circuits.

## Figures and Tables

**Table 1:** Summary of the measured results for the synchronous rectifiers compared to the previous works.

Ref.	Device (Part Number)	Circuit Topology	Freq. (GHz)	Eff. (%)	Output dc Power (W)	Coupler	Phase Shifter
[18]	GaN HEMT (CGH40010F)	Class-F	0.985	81.3	8.7	External	External
[19]	GaN HEMT (CGH40010F)	Class-$F^{-1}$	0.6–1.15	80.1	8	External	External
[20]	GaN HEMT (CGH40010F)	Class-$F^{-1}$	1.8	77	6.9	External	External
[21]	GaN HEMT (CGH40010F)	Class-$F^{-1}$	1.17	78	5.1	Integrated on module	Integrated on module
		Class-F	2.4	75.5	7.18		
This work	GaN HEMT (CGH40006P)	Class-F, independent second harmonic tuning circuit	2.4	69.6	7.81	Integrated on module	Integrated on Module

## Data Availability

The datasets involved in this paper are all public datasets and have been appropriately cited.
